# Comment on ‘low-density lipoprotein cholesterol and clinical outcomes in patients with liver cirrhosis: a nationwide cohort study’

**DOI:** 10.1080/07853890.2025.2588694

**Published:** 2025-11-17

**Authors:** Yinying Chai, Tinghui Xu, Shengliang Qiu

**Affiliations:** The First Affiliated Hospital of Zhejiang Chinese Medical University (Zhejiang Provincial Hospital of Chinese Medicine), Hangzhou, Zhejiang Province, China

Dear Editor

We read with great interest the study by Kim et al. examining the relationship between low-density lipoprotein cholesterol (LDL-C) levels and clinical outcomes in liver cirrhosis (LC) patients [1]. This large-scale, nationwide cohort study offers valuable insights into lipid profiles and their impact on the prognosis of this high-risk group. We commend the authors for their thorough analysis and the significant contribution to the field of lipid management in LC. However, we respectfully present a few suggestions that could further refine future studies.

First, the study relies on the Friedewald formula to calculate LDL-C, which may lead to inaccurate estimations in LC patients. The formula assumes a constant triglyceride-to-very-low-density lipoprotein cholesterol (TG:VLDL-C) ratio of 5:1, which actually varies significantly across the range of triglyceride and cholesterol levels [2]. This phenomenon is even more pronounced in LC patients. Liver damage in cirrhosis impairs VLDL synthesis and secretion, potentially lowering triglyceride levels or altering VLDL structure, which disrupts the TG:VLDL-C ratio [3]. More accurate methods, such as ultracentrifugation, directly measure LDL-C and provide more reliable results in such patients [2].

Second, liver cirrhosis-induced abnormalities in lipoprotein structure and function [4], which could potentially weaken the association between conventional LDL-C measurements and atherosclerotic risk. Future studies should explore how cirrhosis alters LDL and HDL metabolism and consider including markers of oxidized lipoproteins to improve cardiovascular risk stratification in this population.

Third, the subgroup analyses face challenges related to small sample sizes, particularly in group with LDL-*C* ≥ 190 mg/dL (*n* = 3,555). Further stratification would reduce sample sizes, leading to potential underpowering and an increased risk of type II errors (false negatives). We recommend using advanced statistical methods, such as Bayesian hierarchical models, to pool data across subgroups while accounting for heterogeneity. Alternatively, multi-centre collaborations or larger sample sizes could ensure sufficient statistical power for subgroup analyses.

Fourth, the study performs multiple hypothesis tests, which increases the risk of multiple comparisons bias, particularly in smaller subgroups. To mitigate this, we suggest applying corrections such as the Bonferroni correction or False Discovery Rate (FDR) adjustments to control type I errors and ensure that observed associations are not due to random chance.

Finally, while the study accounts for baseline comorbidities, it does not consider how these conditions may change over time, such as the onset of new hypertension or diabetes or changes in treatment regimens. These dynamic factors could significantly affect both LDL-C levels and clinical outcomes. We recommend incorporating follow-up data on comorbidities and treatments, applying time-dependent Cox regression models [5], and integrating case management by medical social workers. Regular home visits, telephone follow-ups, and updates to electronic health records could provide more comprehensive and real-time data to reduce residual confounding.

Our suggestions aim to enhance this already excellent work. We believe that incorporating these refinements could improve the accuracy and clinical interpretability of LDL-C-related risk stratification, ultimately helping to guide more personalized management strategies for cirrhotic patients. We hope these points will be considered in future studies to refine the assessment of LDL-C-related risks and outcomes in this high-risk population.

## Data Availability

No datasets were generated or analyzed in this study.
